# Exclusive Breastfeeding in Health Personnel: Incidence and Barriers

**DOI:** 10.3390/children10081424

**Published:** 2023-08-21

**Authors:** Tongta Nanthakomon, Sonthaya Nukaw, Sudatip Kositamongkol

**Affiliations:** 1 Department of Obstetrics and Gynecology, Faculty of Medicine, Thammasat University, Pathumthani 12120, Thailand; ntongta@gmail.com; 2 Lactation Clinic, Outpatient Department Thammasat University Hospital, Pathumthani 12120, Thailand; kaengt@hotmail.com; 3 Department of Pediatrics, Faculty of Medicine, Thammasat University, Pathumthani 12120, Thailand; 4 Thammasat University Center of Excellence in Modern Technology and Advanced Manufacturing for Medical Innovation, Thammasat University, Pathumthani 12120, Thailand

**Keywords:** exclusive breastfeeding, health personnel, breast feeding

## Abstract

Exclusive breastfeeding for 6 months (EBF) in healthcare personnel is challenging due to work schedules, high workloads, or separation issues. This study aimed to evaluate the incidence and factors related to EBF in our hospital personnel. Material and Methods: This was a cross-sectional study. Female employees who took maternity leave within 2 years were approached. A questionnaire regarding factors associated with EBF was sent to participants. Factors associated with EBF were analyzed using logistic regression analysis. Results: There were 110 mothers enrolled. The mean maternal age was 32.5 ± 4.21 years, 66.36% came from the nursing department, the infant’s age was 6–24 months, and 46.4% of mothers had previous breastfeeding experience. Our EBF for 6 months rate was 63.6%. Breastfeeding attitude (OR = 1.12, 95%CI 1.08–1.38), perception of breastfeeding obstacle (OR = 1.45, 95%CI 1.26–1.66), breastfeeding behavior (OR = 1.17, 95%CI 1.08–1.26), and support from health system (OR = 1.09, 95%CI 1.01–1.19) were significantly associated with EBF. From multiple logistic regression models, perception of breastfeeding obstacles (aOR 1.55, 95%CI 1.27–1.90), breastfeeding behavior (aOR 1.12, 95%CI 1.01–1.24), and support from health care system (aOR 0.84, 95%CI 0.72–0.97) remain the significant factors associated with successful EBF. Conclusion: Successful EBF was prevalent in mothers who had good attitudes to breastfeeding, perceived low levels of obstacles, and had support from the health care system.

## 1. Introduction

Breastfeeding is widely accepted as the most proper way to feed infants. The health benefit of breastfeeding has been well demonstrated in both infants and mothers. For infants, breastfeeding can reduce infant mortality rate, hospital admission, and respiratory and gastrointestinal infection. A study from lower-middle-income countries found lower mortality due to infectious disease in exclusively breastfed infants compared to infants with predominant breastfeeding [1]. This protective effect was also evident in older children; the same study had reported lower mortality from infectious disease in infants less than 2 years, who received any breastmilk compared to infants who had not received breastmilk [2]. Admission from respiratory tract infection during the first 2 years of life was also reduced in more versus less breastfeeding [3]. The health benefits of breastfeeding are not limited to the early infancy period. There was a meta-analysis that reported a 26% reduction in the odds of being overweight or obese [4]. Long-term health benefits of breastfeeding can be explained through the positive effect of breastmilk on infant gut microbiota. At birth, the gut microbiota is immature, and it will be shaped through the early childhood period. Genetics, mode of delivery, breastmilk feeding, type of complementary feeding, and antibiotic exposure play important roles in gut microbiota shaping. A healthy gut microbiome is evident in reducing metabolic diseases such as type 2 diabetes, obesity, and hypertension in adult life. For mothers, the longer duration of breastfeeding protected women from breast cancer and ovarian cancer [5]. In addition to health benefits, breastfeeding had significant economic effects World Health Organization (WHO)/The United Nations international children’s emergency fund (UNICEF) recommends exclusive breastfeeding for the first 6 months and continued breastfeeding with adequate complementary food for up to 2 years and beyond. The Lives Saved Tool reported 823,000 annual deaths of children under 2 years would be saved if breastfeeding was scaled up [6]. For Southeast Asia countries, optimal breastfeeding can save 262 infants’ deaths annually, saving 7.65 million USD/year for healthcare expenses [7]. In 2025 WHO intends to increase the rate of exclusive breastfeeding (EBF) for the first 6 months to at least 50% [8]. However, many obstacles endanger the duration of breastfeeding. Our national data from Multiple Indicator Cluster Surveys (MICS-2019) shows that exclusive breastfeeding for 6 months in Thailand was 14% compared to 23.1% in 2016 [9]. This finding raised awareness of the importance of promoting breastfeeding in our population. There are many factors associated with premature cessation of breastfeeding in Thailand such as cultural traditions to give water to newborns, antenatal breastfeeding education, and maternal employment. Among these obstacles, maternal employment is one of the most common barriers to EBF worldwide [10,11,12,13]. Workplace accommodation for breastfeeding was found effective in improving the breastfeeding rate [14,15,16]. Physician’s breastfeeding behavior is the strongest predictor for breastfeeding advocacy [17] and physician breastfeeding advice has a positive impact on breastfeeding initiation and continuation [18]. In fact, healthcare employees face more complicated problems due to working schedules, high volumes of workloads, and separation issues [15,19,20]. In low-middle-income countries, the problem might be more severe because of the low staff-patient ratio and high working hours. Some countries have workplace policies to support breastfeeding such as prolonged maternity leave or delayed night-shift after returning to work. Nowadays, Thailand has no regulation to support breastfeeding in health care personnel. Therefore, continuation of breastfeeding after returning to work in our health care personnel is even harder. Ensuring that staff have sufficient knowledge, competence, and skills to support breastfeeding is one of the critical management steps in “The ten steps to successful breastfeeding” [21]. Successful EBF in hospital personnel is the best indicator of their knowledge and skills. Therefore, in order to improve the patient breastfeeding rate, supporting breastfeeding in our healthcare personnel should be one of the most important strategies. Our hospital is a university-based hospital that rendered more than 4000 births per year. Currently, we rarely have any breastfeeding support policy for our staff. This study was the starting point to facilitate breastfeeding support in our hospital. For the effectiveness of the upcoming breastfeeding campaign, the main purpose of this research was to assess the success rate of EBF for 6 months in hospital personnel and factors associated with successful breastfeeding.

## 2. Materials and Methods

This study was a cross-sectional descriptive study conducted in a university hospital in Pathumthani, Thailand. Inclusion criteria were female hospital employees who had maternity leave from January 2019–December 2020 and who had their youngest child aged between 6 months to 2 years. Subjects were excluded if mothers or infants had contraindications to breastfeeding such as HIV infection, using chemotherapeutics agents, or inborn errors of metabolism. This research has been approved by The Human Research Ethics Committee of Thammasat University Hospital.

### 2.1. Sample Size Calculation 

Based on our data from the Human Resources Department, we have a total of 153 hospital employees who had maternity leave from January 2019–December 2020. From Krejcie and Morgan’s 1970 formula, in order to achieve a 95% confidence interval with a degree of accuracy at 0.05, and a population proportion of 0.5, we needed a total of 110 subjects to participate in this study [22].

### 2.2. Methods

Based on our human resource data, mothers who met inclusion criteria were approached individually to have the study explained and written informed consent was obtained. Enrolled subjects were asked to complete the questionnaire using an electronic form. The questionnaire was divided into 6 parts as follows:

1. Mothers’ and infants’ baseline characteristics were obtained from 16 questions including age of mothers and infants, marital status, education, income, family type, working schedule, duration of maternity leave, breastfeeding experience, prenatal breastfeeding education, and separation of mothers and infants after returning to work.
2. Maternal attitudes toward breastfeeding. There were 12 questions, a 4-point Likert -scale, which contained both positive and negative attitudes toward breastfeeding. The interpretation of attitudes score was divided into 3 levels: positive attitudes (35.1–48), neutral (32.2–35), and negative attitudes (15–32.1). A higher score indicated more positive attitudes toward breastfeeding. This questionnaire was verified with Cronbach’s coefficient alpha approach of 0.82 [23].
3. Obstacles to breastfeeding. There were 10 questions (4-point Likert type) with a total score of 40 where higher scores mean fewer obstacles. The questions focused on how often the mother experiences these obstacles to breastfeeding. She was asked to answer how often this obstacle occurs to her, such as pain in feeding, feeling tired, not producing enough milk, cracked nipples, or breast pain. The 4-point Linkert scale was very often, often, occasionally, and never. The interpretation of obstacles to breastfeeding was divided into 3 groups as follows: High-level obstacles (14.6–22.3), intermediate obstacles (22.4–30.7), and low-level obstacles (30.8–39). Lower scores meant mothers experienced more obstacles. This question was verified with Cronbach’s coefficient alpha of 0.72 [23].
4. Breastfeeding behaviors. This part focused on maternal breastfeeding behavior regarding preparation for breastfeeding, methods of feeding, milk expression, preparing for child caregiver and source of support, storage, and preparation of milk when returning to work, problem-solving skills regarding breastfeeding issues. This part had 20 questions with a total score of 60, with higher scores meaning better breastfeeding behaviors. This set of questions was verified with Cronbach’s coefficient alpha of 0.87 [24].
5. Environmental factors contributing to breastfeeding. This set had 10 questions with a 4-point Linkert scale. This part focused on accessibility to health services for pregnant and postpartum women such as lactation clinics, breastfeeding support in the early postpartum period, community support for lactating mothers, and support from the workplace. A higher score meant that she felt more supported. This set of questions was verified with Cronbach’s coefficient alpha of 0.83 [23].
6. Workplace breastfeeding support. We have 2 open-ended questions. The first question was “What support you have during your breastfeeding period”? The second was “What support you would like to have during breastfeeding”? The participants were asked to narratively explain workplace support they already have and support they would want to have in the future.

The baseline characteristics questionnaire was developed by our team. The questionnaire regarding maternal attitude toward breastfeeding, the obstacle to breastfeeding, and environmental factors contributing to breastfeeding was developed by Chuprapan et al. [23]. These questionnaires had undergone content validation by experts and received a content validity index of 0.82, and Cronbach’s coefficient was varied as mentioned above. The breastfeeding behavior questionnaire was derived from the study of Rungreang K. and the questionnaire was reviewed by experts in breastfeeding, receiving a content validity index of 0.82 [24].

EBF was defined as infants receiving only breastmilk, no other solids or liquids including infant formula or water, except for medication, vitamin and mineral supplementation [25].

### 2.3. Statistical Analysis 

Data were analyzed using STATA 14.0. The dependent variable was exclusive breastfeeding at 6 months. Independent variables were other factors associated with successful EBF such as education, income, work schedule, attitude toward breastfeeding, and perception of breastfeeding obstacles, etc. Categorical variables were described in frequency and percentage and analyzed using an exact probability test. For continuous variables, data were tested for normal distribution by the Shapiro–Wilk test. Normally distributed variables were presented in mean (standard deviation; SD) and analyzed using a *t*-test. Non-normally distributed data were presented in median (interquartile range; IQR) and analyzed using a rank sum test. Variables were considered significant if the *p*-value < 0.05. Simple logistic regression was used to screen variables associated with successful EBF. Factors in which the *p*-value from the univariable analysis was less than 0.1 were included in the multivariable analysis. Multiple logistic regression models underwent a goodness-of-fit test.

## 3. Results

**Figure 1:**
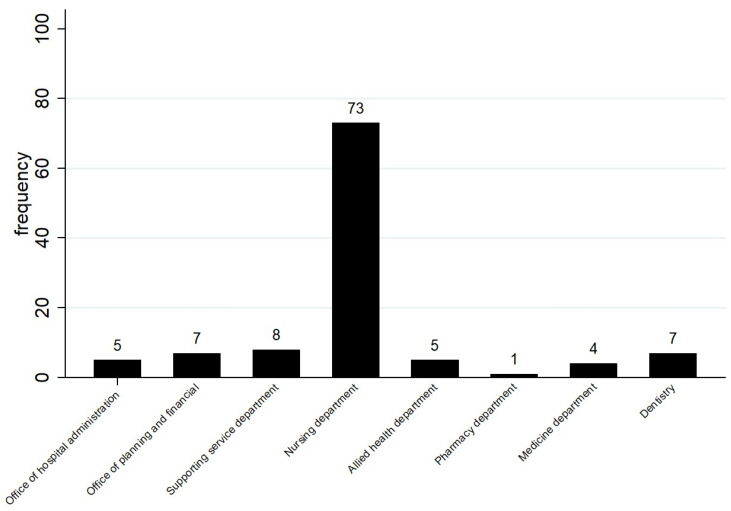
Distribution of subject’s affiliation.

There were 110 mothers enrolled in the study. Exclusive breastfeeding for 6 months was found in 70 subjects (63.7%). Mother’s age ranged from 21–43 years; infant’s age ranged from 6–24 months. Most of the subjects came from the nursing department (73, 66.4%). The distribution of the subject’s affiliation is shown in Figure 1.

Most of them were married (101, 91.9%), and received a bachelor’s degree or higher education (77, 70%). The majority of subjects (74, 67%) worked shifts, and only 36 mothers (32.7%) had a work schedule only from 8 a.m. to 4 p.m. The baseline characteristics of subjects are shown in Table 1.

As shown in Table 2, mothers in both successful EBF and those who did not, had positive attitudes toward breastfeeding, but mothers in the EBF group had better scores compared to mothers who did not (*p* = 0.002). This meant that mothers who were successful had a more positive attitude toward breastfeeding. As well as obstacles to breastfeeding, mothers in the EBF group had higher scores than mothers who did not. (*p* < 0.001). This implied that mothers who did not succeed experienced problems in breastfeeding more than mothers who did. Regarding breastfeeding behaviors, mothers who succeeded also had better scores than mothers who did not (*p* < 0.001). Although all mothers were hospital personnel, there were some differences in the support they received. Mothers who succeeded with EBF reported less support from environmental factors contributing to breastfeeding. (*p* = 0.081).

Simple logistic regression showed an association between successful EBF and attitudes toward breastfeeding, perception of breastfeeding obstacles, breastfeeding behavior, and Environmental factors contributing to support of breastfeeding, *p* < 0.05. Marital status, education, family type, mother’s income, type of work, duration of maternity leave, breastfeeding experience, times of prenatal breastfeeding education, and separation from infants at 3 and 6 months were not significant factors contributing to EBF as shown in Table 3.

Multiple logistic regression model included previous breastfeeding experience, receiving prenatal education more than 2 times, maternal attitudes score, perception of breastfeeding obstacles, breastfeeding behavior, and support from the health care system, which had *p* < 0.1 from simple logistic regression, Goodness-of-fit test was used to test the model, *p* = 0.24.

From the multiple logistic regression model, perception of breastfeeding obstacles and breastfeeding behavior remain the significant factors associated with successful exclusive breastfeeding as shown in Table 4. Surprisingly, we found that mothers in the success group have less environmental support than mothers who did not (*p* = 0.019).

Regarding workplace breastfeeding support, all mothers answered about the support they had already had but only 69 mothers answered about the support they would want to have in the future. Regarding support they already have, the answer can be categorized into 1. Access to a clean refrigerator to store milk (*n* = 9). 2. Break-time to express milk (*n* = 61). There were 40 (36%) mothers who reported that they did not receive any support from the hospital or colleagues. For support, they would want to have in the future can be categorized into 3 categories. 1. Access to clean refrigerator and designated space to express milk (*n* = 11) 2. Need for official breastfeeding break time (*n* = 27). 3. Postponing the night shift (*n* = 2). There were 29 (26%) mothers who reported that they already had good support from colleagues.

## 4. Discussion

Maternal employment is the major factor for premature breastfeeding cessation worldwide. A study from a northern province in Thailand reported maternal employment has a negative impact on breastfeeding continuation beyond 1 year. Interestingly, all types of working schedules including fixed schedules, rotational schedules (shift works), or even mothers who have work-from-home jobs had a breastfeeding rate of 1 year which was lower than stay-at-home mothers [26]. In this study, even though all mothers were employed, our exclusive breastfeeding rate at 6 months was 67.6%, which was much better than 14% from national survey data by UNICEF in 2019 (MICS-2019) [9]. This implied that even with separation issues, stress from work, and rotational work schedules, most of our mothers thrived with breastfeeding. However, our subjects might have some differences from mothers in MICS. Most of our participants received a bachelor’s degree or higher and more than 70% of our mothers had an average monthly income of at least 30,000 Thai baht (around 916 USD) whereas the average monthly income per household in Thailand in 2020 was 27,000 Thai baht (825 USD). In Thailand, paid maternity leave is 45 days, and can be extended to 90 days without pay for the latter half. Social welfare will grant maternity leave allowance at the rate of 50 percent of the average salary for 90 days. Most mothers in our study returned to work after 90 days of maternity leave, which is much longer than a mother from a lower-income family with no social welfare who relies on a daily wage and was therefore required to return to work earlier. A study in Thailand reported more than 70% of mothers had returned to work earlier than 90 days. The main reasons for returning to work earlier were earnings, and fear of affecting salary increments or bonuses [27]. Furthermore, this study was carried out on hospital personnel, therefore participants should have better knowledge of breastfeeding benefits both for mothers and children. This encouraged them to push more effort to breastfeed. They also know more about how to breastfeed, and it is easier to get help if facing problems.

Comparing mothers who succeeded and who did not, there was no statistical difference in most of the baseline characteristics such as education, family type, income, and marital status between mothers who did or did not EBF. This could be explained by this study being executed in a single hospital where most of the subjects had similar characteristics. Shift work had been reported to negatively affect breastfeeding [28]. In Thailand, there are no regulations regarding resuming the night shift after returning to work. In Turkey, mothers had 16 weeks of maternity leave and unpaid leave for up to 24 months. A prior study in physician mothers from Turkey showed that the exclusive breastfeeding duration was 4.8 months and the total breastfeeding length was 15.8 months. The most common reason for weaning was workplace-related conditions. The mean time of resuming the night shift was 8.6 months [19]. Since 70 mothers (67%) in our study did shift work and we do not provide childcare service in our hospital, this might explain why 36 mothers (33%) needed to separate from their infants after returning to work, which can endanger breastfeeding. However, we did not find any difference in EBF between mothers who were separated from their infants and those who were not.

A study focusing on breastfeeding in employed mothers who work shifts revealed that shift-work mothers had a lower rate of breastfeeding compared to non-shift-work mothers and factors contributing to successful EBF and continuation of breastfeeding beyond 6 months of life were the use of lactation room and breast-pumping break in both shift and non-shift work groups [28]. Contrary to our study, the EBF rate was not different between mothers who worked shifts and mothers who did not. However, because most of our participants worked shifts, therefore, this study would be under power to investigate the effect of shift work on breastfeeding duration. Unfortunately, our hospital has no designated lactation room or policy of delayed shift work or official breast-pumping breaks, so we did not have data regarding workplace lactation support on breastfeeding duration.

Multiple logistic regression had shown that perception of breastfeeding obstacles, and breastfeeding behavior had a significant effect on the success rate. The obstacle to breastfeeding in our study meant how often the mother experienced problems during breastfeeding such as feeling tired, nipple pain cracked nipple, or feeling of not having enough milk. This can be alleviated by prenatal breastfeeding education either lactogenesis process, how to breastfeed, correct position and attachment, and good immediate postpartum management. Breastfeeding behaviors such as how to feed infants and how to prepare milk for infants during separation, were different between mothers who succeeded and mothers who did not. These findings supported the theory of breastfeeding education focusing on self-efficacy using the adult learning model [29,30]. Based on Bandura’s theory [31], self-efficacy refers to an individual’s belief in his or her capacity to execute behaviors necessary to produce specific performance attainments. This is not only related to knowledge, as our study showed that most of our subjects had a high score in breastfeeding attitude, but the difference came from perception of obstacles, and more appropriate breastfeeding behaviors. Mothers who had the intention and “perceived capability” to breastfeed were more likely to overcome challenges during breastfeeding [16]. If the mother had self-efficacy, she would have the confidence to overcome challenges that may happen after returning to work such as separation issues, milk-expression-related problems without difficulties, and perceive a low level of breastfeeding obstacles. This attitude should be emphasized during prenatal education and early postpartum breastfeeding support. Even though our mothers were all working in a hospital, 30% reported receiving prenatal breastfeeding education less than standard prenatal care in Thailand (2 times). This might come from a tight working schedule, or they did not recognize the importance of prenatal education. This should be our future area of development. Unexpectedly, we found mothers who succeeded had less support from public health and their environment. This might be explained by successful mothers feeling less problem or having better knowledge that helped them succeed in breastfeeding even with less support.

Currently, our hospital has no official lactation accommodation program. Lactation support had been varied due to work characteristics, supervisor, and colleagues. As described earlier, workplace accommodation to support breastfeeding has a positive effect on breastfeeding continuation [14,32,33]. Mothers who received lactation support services based on the 2012 affordable care act (ACA) had increased breastfeeding duration and duration of exclusive breastfeeding [33]. Data from a national survey in the USA reported only 40% of women giving birth during 2011–2012 had access to breastfeeding break time and private space. In women who had access, they had 2.3 times to EBF at 6 months [14]. Most of our participants had a problem expressing milk during work. Even in mothers who got support from peers/supervisors. They also felt uncomfortable leaving for milk expression. Breastfeeding break was the most wanted lactation support among our participants, followed by access to dedicated space and a clean refrigerator to store milk. Surprisingly, nobody asked for daycare in the hospital which used to be a measure to support breastfeeding in the workplace.

The main limitation of our study was that since it was a single-center study, there was less variety of subjects. Most of the participants were nurses. We were unable to show adequate support that should be provided in the workplace to make EBF easier. Enhancing the level of lactation support among hospital employees should be the next move in our hospital, in addition to improving prenatal breastfeeding counseling.

## 5. Conclusions

Our study has heightened the significance of prenatal breastfeeding education focused on competencies to overcome obstacles and maternal breastfeeding behavior.

## Figures and Tables

**Table 1:** Baseline characteristics, socioeconomics and factors contributing to exclusive breastfeeding.

Factors	Not Exclusive Breastfeeding	Exclusive Breastfeeding	*p*-Value
	N = 40	N = 70	
Mothers’ age (y) ^1^	32.3 ± 4.4	32.6 ± 4.2	0.661
Infants’ age (months) ^2^	16.5 (9, 21.5)	16 (10, 22)	0.8615
Marital status (*n*, %)			
Married	35 (87.5)	66 (94.3)	0.281
Single	5 (12.5)	4 (5.7)	
Maternal education (*n*, %)			
Less than bachelor	15 (37.5)	18 (25.7)	0.203
Bachelor or more	25 (62.5)	52 (74.3)	
Maternal income (thb/month) (*n*, %)			
30,000 or less (≤916 USD)	12 (30)	19 (27.1)	0.827
More than 30,000 (>916 USD)	28 (70)	51 (72.9)	
Family type (*n*, %)			
Single	15 (37.5)	31 (44.3)	0.550
Extended	25 (62.5)	39 (55.7)	
Working schedule (*n*, %)			
8 a.m.–4 p.m.	15 (37.5)	21 (30)	0.527
Shift work	25 (62.5)	49 (70)	
Duration of maternity leave (*n*, %)			
Less than 90 days	2 (5)	6 (8.6)	0.708
90 days or more	38 (95)	64 (91.4)	
Breastfeeding experience (*n*, %)			
No	26 (65)	33 (47.1)	0.078
Yes	14 (35)	37 (52.9)	
Prenatal breastfeeding education (*n*, %)			
Less than 2 times	16 (40)	17 (24.3)	0.090
2 times or more	24 (60)	53 (75.7)	
Separation from infants before 3 months of age (*n*, %)			
Yes	1 (2.5)	2 (2.9)	1.000
No	39 (97.5)	68 (97.1)	
Separation of infants between 4–6 months of age (*n*, %)			
Yes	14 (35)	22 (31.4)	0.833
No	26 (65)	48 (68.6)	

^1^ Data were described in mean ± SD and analyzed using *t*-test. ^2^ Data were described in median (IQR) and analyzed using rank-sum test.

**Table 2:** Breastfeeding attitude, obstacles, behavior, and health system support scores.

Factors	Not Exclusive Breastfeeding	Exclusive Breastfeeding	*p*-Value
	N = 40	N = 70	
Breastfeeding attitudes score	41 (38, 45)	43.5 (41, 46)	0.002
Obstacles to breastfeeding	32 (29, 35.5)	38 (36, 40)	<0.001
Breastfeeding behavior	47 (39.5, 50)	51.5 (48, 54)	<0.001
Environmental factors contributing to support of breastfeeding	33.5 (29.5, 37)	36 (32, 39)	0.081

Data were presented in median (IQR), analyzed with a rank-sum test.

**Table 3:** Simple logistic regression analysis for factors associated with exclusive breastfeeding.

Factors	Odds Ratio	95% Confidence Interval	*p*-Value
Married	0.424	0.11–1.68	0.222
Education bachelor or higher	1.73	0.75–3.99	0.197
Extended family type	0.755	0.340–1.67	0.488
Income > 30,000	1.150	0.49–2.71	0.749
Shift work	1.4	0.62–3.18	0.421
Maternity leave ≥ 90 days	0.561	0.11–2.92	0.493
Has breastfeeding experience	2.082	0.93–4.64	0.073
Received breastfeeding education ≥ 2 times	2.078	0.90–4.79	0.086
Not separate with infants before 3 months	0.872	0.08–9.93	0.912
Not separate with infants between 4–6 months	1.175	0.52–2.67	0.701
Breastfeeding attitude	1.122	1.08–1.38	0.001
Perception of breastfeeding obstacles	1.445	1.26–1.66	<0.001
Breastfeeding behavior	1.165	1.08–1.26	<0.001
Environmental factors contributing to support of breastfeeding	1.092	1.01–1.19	0.039

**Table 4:** Multiple logistic regression analysis for factors associated with exclusive breastfeeding.

Factors	Adjusted Odds Ratio	95% Confidence Interval	*p*-Value
Has breastfeeding experience	1.42	0.48–4.19	0.992
Received breastfeeding education ≥ 2 times	0.99	0.30–3.32	0.520
Breastfeeding attitude	1.09	0.92–1.29	0.299
Perception of breastfeeding obstacles	1.55	1.27–1.90	<0.001
Breastfeeding behavior	1.12	1.01–1.24	0.022
Environmental factors contributing to support breastfeeding	0.84	0.72–0.97	0.019

## Data Availability

Data is unavailable due to privacy.
